# Diseases of Infants and Children

**Published:** 1910-01

**Authors:** 


**Diseases of Infants and Children.** By **Henry Dwight Chapin,** A.M., M.D., Professor of Diseases of Children, New York Post-Graduate Medical School and Hospital. And **Godfrey Roger Pisek,** M.D., Professor of Diseases of Children, University of Vermont. Octavo, pp. 624. With 179 illustrations and 11 colored plates. New York: William Wood & Co. (Cloth, $4.50 net.)

This is an exceptionally well written and practical work on a most important subject. Professor Chapin and his associate, Professor Pisek, are considered authorities in this department of medicine, as both have for many years occupied leading places as teachers of pediatrics. The style of the book is clear, the teaching plain, and the element of personal experience well marked throughout.

The suggestion scheme for diagnosis is well arranged and helpful, the chapters on infant feeding excellent and complete. A truism is the statement on page 99, that food "may be chemically right but practically wrong" and that "each infant is a law unto itself except concerning the form in which it prefers its food." The book can be recommended as a valuable addition to pediatric literature. J. A. R.

